# Ovulation induction techniques in women with polycystic ovary syndrome

**DOI:** 10.3389/fmed.2022.982230

**Published:** 2022-08-12

**Authors:** Andreas A. Vyrides, Essam El Mahdi, Konstantinos Giannakou

**Affiliations:** ^1^Barts and the London School of Medicine and Dentistry, Queen Mary University of London, London, United Kingdom; ^2^Department of Obstetrics and Gynecology, Newham University Hospital NHS Trust, London, United Kingdom; ^3^Department of Health Sciences, School of Sciences, European University Cyprus, Nicosia, Cyprus

**Keywords:** polycystic ovary syndrome, Ovulation Induction, PCOS (polycystic ovarian syndrome), polycystic ovarian disease, infertility

## Abstract

Anovulation is very common and has several different clinical manifestations, including amenorrhea, oligomenorrhea and abnormal uterine bleeding. Various mechanisms can cause anovulation. The clinical consequences and commonest chronic anovulatory disorder, polycystic ovary syndrome (PCOS), has a prevalence that ranges between 6 to 10% of the global population. While multiple causes can eventually result in PCOS, various methods have been described in the literature for its management, often without ascertaining the underlying cause. Ovulation Induction (OI) is a group of techniques that is used in women with PCOS who are looking to conceive and are unbale to do so with natural means. This narrative review presents a summary of the current evidence and available techniques for OI in women with PCOS, highlighting their performance and applicability.

## Introduction

Among the classification criteria for stratifying anovulatory women, normogonadotropic normoestrogenic patients, also known as World Health Organization (WHO) group II, account for around 80% of all anovulatory patients (1). Polycystic Ovary Syndrome (PCOS) is the most common disorder affecting this subgroup of patients, with prevalence ranging from 6 to 10% of the global population depending on the classification criteria used (2). It is recognized that PCOS is not a cause of anovulation but rather a symptom of chronic anovulation caused by a range of both endocrinologic and functional abnormalities (3). As opposed to normal cycling hormone concentrations in ovulating women, women with PCOS are often described as being in a “hormonally steady state,” with both gonadotropin and sex steroid concentrations similar to those seen in early follicular phase (4). Increased Luteinizing Hormone (LH) levels are a result of abnormal LH secretory dynamic due to increased LH pulse frequency and pulse amplitude (5–8), where decreased FSH levels results from the increase in Gonadotropin Releasing Hormone (GnRH) pulse frequency, the negative feedback effects of chronically elevated estrone concentrations and increased inhibin B levels (9, 10). These changes often result in an increased LH/FSH ratio (11–13).

While menstrual irregularities, and by association infertility, are the most common complaints in women with PCOS, they are seldom isolated findings and are accompanied by an increase in both androgen and estrogenic hormone production. Elevated levels of serum testosterone, androstenedione, and 17α-hydroxyprogesterone have been identified to be LH dependent, derived mainly from the ovaries, while levels of dehydroepiandrosterone (DHEA), dehydroepiandrosterone sulfate (DHEA-S), and estrone are mainly derived from the adrenals (14–16). High concentrations of androstenedione are converted into estrone by peripheral tissues, while FSH and oestradiol levels are relatively stable and comparable with levels of a normal follicular phase cycle (4). A positive feedback of increased ovarian androgens contributes to insulin resistance which in turn inhibits hepatic sex hormone binding globulin production, further increasing androgen levels (17–19). These changes are responsible for the pathophysiological symptoms of PCOS, such as insulin resistance, which predisposes to type 2 diabetes, obesity, hirsutism, and anovulation (3). A stepwise approach in treating and managing patients diagnosed with PCOS can range from lifestyle interventions to invasive operations such as Laparoscopic Ovarian Drilling (LOD), which is often reserved for medication resistant cases. The present narrative review summarizes the available evidence and current techniques commonly used in clinical practice for Ovulation Induction (OI) in women with PCOS. Table 1 summarizes the approaches listed in this review.

**Table 1:** Summary of available Ovulation Induction (OI) techniques.

**Intervention**	**Supplementary drug**	**OI Initiation** **(Day of Cycle)**	**Protocol**	**Protocol progression**	**Protocol monitoring**	**Expected result**
Weight loss and lifestyle modifications	–	–	Loss around 5–10% of body weight (20–26)	–	–	•Reduce hyperinsulinemia, (27–31) • Increase insulin sensitivity, (27–31) • Restore ovulatory cycles, (27–31) • Improve reproductive outcomes including ovulation and menstrual cycle regulation (32).
Myo–Inositol	–	–	–	–	–	• Improving insulin sensitivity (33) • Increasing Sex Hormone Binding Globulin (SHBG) (33) • Decrease free Testosterone (33) • minimizing hyperandrogenic features (33). • Increase ovulation rates when compared with placebo or no treatment (33)
Clomiphene	–	Day 2–5	50 mg OD for 5 days–traditional	Progestin prescribed for lack of ovulation and cycle restart Increase by 50 mg for each cycle thereafter until response–Upper limit at 250 mg.	Serum progesterone levels, > 3 ng/mL between days 22 and 25 indicates successful ovulation	• Successful in 70–80% of women (34, 35) • Cumulative pregnancy rates of 70–75% are expected over 6–9 cycles of treatment (36, 37)
	–		50 mg OD for 5 days–“stair step”	Increase by 50 mg if lack of dominant follicle on ultrasound	Ultrasound sonography day 11–14, Repeat ultrasound 1 week after dose increase	• Significantly higher ovulation rates of 64% at 100 mg when compared to the traditional 22% at the same dose (38) • Shorter time to ovulation by 32–53 days when compared to the traditional method (38)
	Glucocorticoids	Day 5	Clomiphene 200 mg OD for 5 days Dexamethasone 2mg OD for 10 days	Clomiphene resistant women–no progression	Ultrasound sonography day 16 or 17	• 88% of women had successfully ovulated vs. 20% of in the control group (39) • Cumulative pregnancy rate 40.5% vs. 4.2% in the control group (39)
	Metformin	Day 3	Clomiphene 50 mg OD for 5 days Metformin 500 mg OD–gradually increase to 2g (1g BD)	Increase Clomiphene dose either after 5 weeks of anovulation or after a menses–Upper limit at 150 mg	If 2 consecutive serum progesterone levels > 5ng/mL then weekly pregnancy test until positive or menses occurred	• Clomiphene alone and Clomiphene with Metformin is superior to Metformin alone in live birth rate (40) • Comparable live birth rate in Clomiphene vs. Clomiphene with Metformin (40)
	Myo–Inositol	No available evidence/protocol in the literature for comparison with other protocols				
Letrozole	–	Day 3–5	2.5 mg OD for 5 days	Increase by 2.5 mg for each cycle thereafter until response–Upper limit at 7.5 mg Max 5 cycles for each patient	Mid luteal progesterone >3 ng/mL	• Higher cumulative pregnancy rate (27.3% vs. 21.5%) and higher live birth (27.5% vs. 19.1%)(41) when compared to Clomiphene • Higher proportion of women achieve ovulation (88.5% vs. 76.6%), and a higher proportion of ovulations over total treatment (61.7% vs. 48.3%) when compared to Clomiphene (41)
Exogenous Gonadotropins	–	Day 3–5	75IU hMG/rFSH OD for 5 days–conventional protocol	Increase by 75IU hMG/rFSH until response Triggered with 5,000–10,000 IU hCG	Elevated levels of Estradiol when compared to background Ultrasound sonography for Follicular visualization and triggering	• Cumulative conception rates of around 90% and cumulative live birth rates of 85% after 12 cycles (42) • Risk for OHSS and multifetal pregnancy (43, 44)
	–		37.5–75IU hMG/rFSH OD for 8–14 days–chronic low dose	Increase by 37.5–75IU hMG/rFSH until response Triggered with 5,000–10,000 IU hCG		• Similar cumulative pregnancy and live birth rate with conventional protocol (45–49) • Smaller OHSS and multifetal pregnancy risk than conventional protocol (45–49)
Laparoscopic Ovarian Drilling	–	–	–	Often reserved for medication resistant women–No progression	–	• Similar in live birth rates compared to clomiphene citrate and metformin, gonadotrophins (50). • Lower live birth rates when compared to letrozole (50).

## Weight loss and lifestyle modifications

The prevalence of obesity in PCOS patients varies in different populations, ranging from 42 to 65% (51, 52). While the diagnosis of PCOS does not always indicate an anovulatory state, obese patients are more likely to suffer from it, hindering their ability conceive (53). Modest weight loss around 5–10% of body weight, often restores ovulatory cycles in obese anovulatory women with PCOS (20–26). In a population of PCOS patients, 70–80% of obese patients were found to be insulin resistant while only 20–30% of lean patients were insulin resistant (54). The primary aim in any patient with PCOS would be to promote weight loss through caloric deficit and physical activity, which would reduce hyperinsulinemia, increase insulin sensitivity, and often restore ovulatory cycles (27–31). According to a recent analysis of PCOS weight loss trials, moderate weight loss is associated with the occurrence of sporadic ovulation in a considerable proportion of patients, and that decreases in hyperandrogenism and insulin resistance likely precede any changes in reproductive outcomes (55). The reduction in hyperandrogenism and insulin resistance with or without the reflective reduction in weight loss has also been demonstrated to improve reproductive outcomes including ovulation and menstrual cycle regulation (32). Obesity is a risk factor for poor obstetric outcomes and maternal complications (e.g., spontaneous abortions and thromboembolisms), thus patients should be counseled on maintaining a normal weight throughout pregnancy (53).

## Clomiphine citrate

Acting as a Selective Estrogen Receptor Modulator (SERM), clomiphene has been available for clinical use since 1967 and has been identified to act both as an agonist and an estrogenic antagonist (56–58). As an estrogenic antagonist at the hypothalamic level, it depletes nuclear receptors, therefore indirectly preventing accurate interpretation of circulating estrogen levels, without affecting existing levels. This causes a normal compensatory mechanism by the hypothalamus, altering GnRH secretion, stimulating gonadotropin release and driving ovarian follicular development (56, 59). While the optimal day to begin clomiphene for OI would be day 5 of a cycle, ovulation rates, conception rates and pregnancy outcomes are similar regardless of whether induction begins anytime between day 2 and day 5 (60). Clomiphene dose required for induction is positively correlated with both bodyweight and obesity (61). Despite higher doses required, both obese and lean women achieve similar pregnancy rates (62, 63). There is a reverse association between the likelihood of response to clomiphene and increasing body mass index (BMI) as well as the patient's age (34). While the association between clomiphene and bodyweight for OI has been previously described, to date, there is no laboratory parameter that can precisely predict the required dose for each individual patient (61, 63).

Using the traditional method for OI, treatment is initiated with daily 50 mg tablets for 5 days beginning on the 5th day of the cycle. Even though most women will respond to either 50 mg or 100 mg (64, 65) as per manufacturer recommendations (66), some clinicians have used doses as high as 250 mg (64, 65). As the success rate at doses over 150 mg per day is relatively low, it is often recommended to seek alternative induction methods before using these doses (64, 65). Cycle monitoring for ovulation using the traditional method can be achieved using serum progesterone levels, where levels over 3ng/mL between days 22 and 25 are considered sufficient evidence indicating successful ovulation (67, 68). Whenever ovulation does not occur, a progestin is prescribed to induce menses before the next cycle begins at a higher dose. Induction of ovulation will be successful in 70–80% of women (34, 35) and cumulative pregnancy rates of 70–75% are expected over 6–9 cycles of treatment (36, 37).

A novel “stair step” method uses ultrasound sonography for the identification of successful ovulation while avoiding the use of progestins to induce menses at the end of an unsuccessful cycle (38). Patients are placed on 5 days of clomiphene starting at 50 mg, where ultrasound sonography is used between days 11 and 14 to identify non responders who lacked a dominant follicle. Patients who did not successfully ovulate would be given 5 days of clomiphene at 100 mg, followed with an ultrasound 1 week later. In cases where no follicle was visualized on the 2^nd^ ultrasound, the dose would be increased to 150 mg for 5 days and a 3^rd^ ultrasound would be obtained 1 week thereafter (38). This protocol has demonstrated significantly higher ovulation rates of 64% at 100 mg when compared to the traditional 22% at the same dose (95% CI 45–81, *p* = 0.001) (38). An additional advantage with this method is the time to ovulation was significantly reduced by 32–53 days when compared to the traditional method described above (38).

While the side effects from clomiphene treatment are few and far between mainly due to the short treatment duration, vasomotor symptoms account for around 20%, followed by adnexal tenderness in 5%, nausea in 3%, headache in 1%, and very rarely blurred vision or scotomata (37). The main risk factor to consider would be multifetal pregnancy due to multi follicular development, which is increased to around 7–10% (37, 69–71). Clomiphene's anti estrogenic action on the marginally thinner endometrium is one of its drawbacks in these cycles, but the association between endometrial thickness (EMT) and pregnancy rates has not been elucidated in the literature (72).

## Clomiphene and glucocorticoids

The use of prednisolone and dexamethasone has been described to increase the success rates of OI in clomiphene resistant women. These steroids have been shown to be effective in both continuous treatment, as well as in follicular phase regiments which are often given during days 5 to 14 (39, 73–77). It was initially demonstrated that this method is best suited for women with elevated DHEA-S levels, but further studies revealed that it is also effective in unselected patient groups (39, 73, 74, 78).

A large-scale randomized controlled trial (RCT) with 230 clomiphene resistant women, compared a combined treatment of clomiphene citrate 200 mg daily over days 5 to 9 and dexamethasone 2 mg daily over days 5 to 14 against clomiphene citrate 200 mg and placebo for the same amount of time. In the combined group, 88% of women had successfully ovulated whereas only 20% of women ovulated in the control group. The cumulative pregnancy rate in the combined treatment group was also significantly higher with 40.5 vs. 4.2% in the control group (*p* < 0.0001) (39).

The exact mechanism of which glucocorticoids act on clomiphene resistant women has not been elucidated, although suggested mechanisms include suppression of hyperandrogenism, synergistic actions with FSH's direct effects on oocyte development, and indirect effects of intrafollicular growth factors and cytokines (79). Coadministration of clomiphene and steroids has been argued to be justified for a few cycles and should not be used for extended periods of time to minimize the risks and side effects associated with long term steroid use.

## Clomiphene and insulin sensitizing drugs

Several insulin sensitizing drugs have been investigated for their role in managing PCOS. Metformin acts by reducing hepatic gluconeogenesis, decreasing intestinal glucose absorption, increasing peripheral glucose uptake and utilization, and reducing fatty acid oxidation, resulting in the reduction of circulating insulin levels (80). While treatment with metformin might be considered as an adjuvant for weight management in PCOS patients (81–84) by facilitating weight loss *via* the suppression of appetite (85), the overall effect is modest and often inconsistent. Therefore, it should not be used as an alternative to regular physical exercise and caloric restriction (86, 87).

While metformin and other insulin sensitizing drugs such as myo-inositol has been shown to be effective in increasing ovulation rates in in some women with PCOS (33), a large triple-arm RCT (40) of 626 women with PCOS comparing fertility outcomes between metformin with placebo, clomiphene with placebo, and combined treatment of clomiphene with metformin, concluded that clomiphene is superior to metformin in achieving live birth in infertile women with PCOS (40). They also found no evidence in support for extended release metformin, either alone or in combination with clomiphene citrate, to improve live birth rates in women with PCOS (40). Of interest, adverse event rates in the clomiphene group were comparable to the ones described by other studies mentioned above (37, 69–71).

In a large recent meta-analysis (88), despite the fact that it was limited due to the small number of primary studies with low to medium quality of evidence, there was no observable difference in live birth rates between combined treatment of clomiphene and metformin vs. clomiphene alone, (OR 1.21, 95% CI: 0.92–1.59). However, the authors found gastrointestinal side effects more common in combination therapy (OR 3.97, 95% CI: 2.59–6.08) (88). While the combination therapy group had higher clinical pregnancy rates (OR 1.59, 95% CI: 1.27–1.99) and ovulation rates, (OR 1.57, 95% CI: 1.28–1.92) it suffered from an increased miscarriage rate per woman which was statistically significant (OR 1.59, 95% CI: 1.03–2.46) (88).

Myo-inositol alone or in combination with D-chiro-inositol has been shown to be beneficial in the management of both the endocrine and metabolic profile of women with PCOS, by improving insulin sensitivity and increasing Sex Hormone Binding Globulin (SHBG), leading to binding of Testosterone and therefore minimizing hyperandrogenic features (33). In this extent, it is able to significantly increase ovulation rates in PCOS women when compared with placebo or no treatment (OR 3.57, 95% CI: 1.72–7.45) (88), but its effect on pregnancy and live birth rate has not been elucidated in the literature, nor has it been adequately compared with or against clomiphene (89).

It is evident from the available data that stratification of results using BMI is an important factor which further stresses the importance of lifestyle modifications such as physical activity and diet. The available evidence also suggests that clomiphene use is preferable to metformin for OI in obese women with PCOS due to the improvement of clinical pregnancy and ovulation rates (40), while clomiphene use in combination with metformin is avoided due to a higher risk profile with increased miscarriage rate, against marginal improvements in clinical pregnancy and ovulation rates.

## Aromatase inhibitors

Aromatase is the rate limiting step in the estrogen production in both the periphery and the brain (90, 91). By inhibiting the rate limiting step, thereby decreasing the levels and effect of peripheral estrogen, aromatase inhibitors cause a compensatory increase in the pituitary gonadotropin secretion leading to the development of ovarian follicles (92–94). While this process seems suspiciously similar to that of clomiphene, they vary in that clomiphene blocks central estrogen receptors without changing circulating estrogen levels, whereas aromatase inhibitors restrict estrogen synthesis by blocking its rate-limiting enzyme. Initially it was only indicated for postmenopausal women with breast cancer, but a proof-of-concept study successfully demonstrated letrozole as an effective method for OI in clomiphene resistant women, mainly targeting patients with PCOS and ovulatory infertility (95).

A large double blind, multi-center trial (41), recruited 750 women who were diagnosed with PCOS according to the modified Rotterdam criteria (96), compared the efficacy of clomiphene against letrozole. All patients were randomly assigned on a treatment arm initiated between day 3 and 5 of their cycle after spontaneous menses or withdrawal bleeding induced by medroxyprogesterone acetate, 5 mg per day, for 10 days. Patients were either started on letrozole 2.5 mg for 5 days, or on clomiphene 50 mg for 5 days. In each subsequent cycle, the patients were given up to a maximum of 7.5 mg of letrozole, increasing in steps of 2.5 mg at each cycle, or a maximum of 150 mg of clomiphene increasing in steps of 50 mg at each cycle. Non responders were identified using mid luteal progesterone <3 ng/mL. Patients underwent a maximum of 5 cycles and no additional ovulation trigger was used in either group. The letrozole group had a significantly higher cumulative pregnancy rate (27.3 vs. 21.5%) and significantly higher live birth (27.5 vs. 19.1%, RR 1.44; 95% CI: 1.10–1.87, *p* = 0.007) (41). In addition, the letrozole group had a significantly higher proportion of women achieve ovulation (88.5 vs. 76.6%, RR 1.16; 95% CI: 1.08–1.24, *p* < 0.001), and a significantly higher proportion of ovulations over total treatment (61.7 vs. 48.3%, RR 1.28; 95% CI: 1.19–1.37, *p* < 0.001) (41).

Despite producing a similar estrogenic effect through different mechanisms, one major difference between letrozole and clomiphene citrate is their effect on EMT (97). In a recent meta-analysis (97) targeting WHO group II anovulatory patients and comparing clomiphene with several other OI interventions, concluded that midcycle EMT (WMD −1.39; 95% CI: −2.27 to −0.51) (97) and pregnancy rates (RR 0.78; 95% CI: 0.63–0.95) (97) were both lower in clomiphene groups compared to letrozole groups, even though both groups had comparable ovulation rates (RR 0.97; 95% CI, 0.90–1.04) (97). Results from the same meta-analysis showed no statistical difference in EMT (WMD −0.12; 95% CI: −2.17 to 1.94) and no statistical difference in pregnancy rates (RR 2.44; 95% CI: 0.90–6.66) between the clomiphene group and the 1 mg anastrozole group (97), and no difference in EMT (WMD −1.34; 95% CI: −2.70 to 0.01) and a comparable pregnancy rate (RR 1.36; 95% CI: 0.86–2.15) (97) between clomiphene and tamoxifen groups. Although all drugs compared to clomiphene belong to the same drug class, the current results, while heterogenous, suggest superior pregnancy outcomes when comparing letrozole with clomiphene, whereas no discernible differences were found when using other aromatase inhibitors such as tamoxifen and anastrozole.

## Exogenous Gonadotropins

Initially, from crude urinary extracts used as early as the 1960s (98, 99) containing equal amounts of FSH and LH (100), gonadotropin preparations have evolved over the years into highly purified recombinant preparations that are widely used today (101, 102). They have been shown as effective inducers in both hypogonadotropic hypogonadism (WHO Group I) (103, 104) and PCOS patients (WHO Group II) (105). While it could be argued that human menopausal gonadotropin (hMG/menotropins) should be superior for OI in clomiphene resistant PCOS women when compared with recombinant FSH preparations (rFSH), due to their erratic background FSH and LH secretion, they can be used interchangeably since a meta-analysis of trials comparing their effects found no difference in ovulation rate, pregnancy rate, multiple pregnancy rate and incidence of ovarian hyperstimulation syndrome (OHSS) (105). OI with Gonadotropins is seldom first line due to their less-than-ideal risk profile, which include high rates of multifetal pregnancy, significant risk for OHSS due to the required hCG trigger for ovum release and the higher spontaneous miscarriage rate of 20–25% when compared to background of 15% (43, 44). Despite the fact that there is a statistical linear correlation between patient weight and gonadotropins dose required for response, dosage should be assessed clinically, since no laboratory parameter can precisely predict response threshold for each patient (106).

The conventional protocol consists of daily intramuscular injections (IM) of 75IU of gonadotropins for 5 to 6 days between day 3 and 5 of the cycle, until ovarian response. In the event of non-responders, the dose increases in steps of 75IU. Response is identified by elevated serum Estradiol levels compared to background levels and verified by visualizing follicular development on ultrasound sonography. Targeted ovum size is between 16 and 18 mm, trigged with 5,000 to 10,000 IU of human Chorionic Gonadotropin (hCG) IM.

A “chronic low dose” protocol was developed specifically for PCOS patients who are known to be more sensitive to gonadotropin stimulation, often starting from a lower dose between 37.5 and 75IU. Each cycle would last for 7 to 14 days and subsequent cycles would often increase the dose by 37.5 to 75IU (45–49).

OI with gonadotropins have demonstrated cumulative pregnancy rates of 90% and cumulative live birth rates of 85% after 12 cycles (42). Both protocols have similar live birth rates although “chronic low dose” protocol is preferable due to its main advantages in the reduced rate of multifetal pregnancy and reduced rates of OHSS at the price of longer duration of treatment (45–49).

## Laparoscopic ovariann drilling

The bilateral ovarian wedge resection by Stein and Leventhal in 1935 (96), was the first procedure which brought forth the associations between amenorrhea with the presence of bilateral polycystic ovaries and the underlying hormonal influences which were not the result of inflammatory changes (96). While the same paper describing the wedge resection successfully restored physiologic function of the ovaries with menstruation and allowed two of the seven patients to become pregnant, the procedure fell out of favor with the introduction of clomiphene citrate and the inherent risks associated with invasive operations, such as adnexal adhesions (96).

As newer laparoscopic techniques have been developed, the traditional ovarian wedge technique has transformed to ovarian “drilling,” which aims to use electrocautery or laser vaporization to cause focal destruction of the ovarian stroma in an effort to reduce intraovarian and systemic androgen concentrations (50, 107–111). Despite the fact that LOD may reduce the risk of postoperative adnexal adhesion formation (112–115), a meta-analysis found no statistically significant differences in live birth rates following LOD when compared to clomiphene citrate and metformin (OR 0.59; 95% CI: 0.32–1.09 *p* = 0.09), nor gonadotrophins (OR 0.87; 95% CI: 0.56–1.36; *p* = 0.55) (50). The same study found statistically significant lower live birth rates following LOD only when compared to letrozole (OR 0.55; 95% CI: 0.32–0.92; *p* = 0.02) (50).

## Concluding remarks

Menstrual irregularities, chronic anovulation and infertility are some of the most common complaints of women with PCOS. One of the first and simplest steps in the management of PCOS would be lifestyle modifications such as exercise and caloric restriction, with the goal of lowering the patient's BMI and insulin resistance. This would then further decrease potential complications in the event of a successful pregnancy. Even in the absence of significant weight loss, regular exercise can result in spontaneous ovulation and an enhanced likelihood of a successful OI cycle. Clomiphene citrate is the best known and most widely used ovulation inducing agent, due to its good performance and broad use since its first introduction in 1967. Recent evidence in aromatase inhibitors and more specifically letrozole has shown that it can be more efficacious in OI when compared with clomiphene, with higher cumulative pregnancy rates and higher live birth rates. It could be argued that letrozole should instead be used as a first line treatment as it leads to higher midcycle EMT when compared to clomiphene, although the importance of such measurement remains controversial. Insulin sensitizing agents such as metformin are not suitable for use as OI agents but can be used as adjuvants in lifestyle modifications, aiming to reduce bodyweight, although their results are modest and often inconsistent. Myo-inositol has been shown to Increase ovulation rates in PCOS patients, but no studies have assessed its efficacy on pregnancy and live birth rates. Recombinant gonadotropins, while highly effective, are often reserved for doctors with specialists training and women who have failed all other OI protocols due to their risk involving OHSS, multifetal pregnancy and high spontaneous miscarriage rate. LOD has been reported to be effective in small studies in clomiphene resistant patients, however a meta-analysis of the available data suggests similar live birth rates when compared to clomiphene and metformin cycles and significantly lower live birth rates when compared to letrozole. Clinicians should be able to assess the likelihood of a successful pregnancy outcome in PCOS patients undergoing OI, taking into account age, bodyweight, different protocols used, and duration of infertility. If the foregoing treatments fail to produce a pregnancy, a referral to a specialist fertility clinic for *in vitro* fertilization would be an effective alternative. Future studies should aim to compare the pregnancy outcomes of different OI methodologies described above as well as stratify the effectiveness of LOD against existing medical management.

## Author contributions

All authors listed have made a substantial, direct, and intellectual contribution to the work and approved it for publication.

## Conflict of interest

The authors declare that the research was conducted in the absence of any commercial or financial relationships that could be construed as a potential conflict of interest.

## Publisher's note

All claims expressed in this article are solely those of the authors and do not necessarily represent those of their affiliated organizations, or those of the publisher, the editors and the reviewers. Any product that may be evaluated in this article, or claim that may be made by its manufacturer, is not guaranteed or endorsed by the publisher.
